# Liver Injury and Elevated Levels of Interleukins, Interleukin-2 Receptor, and Interleukin-6 Predict the Severity in Patients With COVID-19

**DOI:** 10.3389/fpubh.2021.778340

**Published:** 2021-12-14

**Authors:** Kuan Luo, Yongfeng Chen, Junjie Yang, Quan Tao, Min Luo

**Affiliations:** ^1^Department of Neurosurgery, Zhongnan Hospital of Wuhan University, Wuhan, China; ^2^Leishenshan Hospital, Wuhan, China; ^3^Department of Neurosurgery, Wuhan Puren Hospital, Wuhan, China; ^4^Department of Management, Zhongnan Hospital of Wuhan University, Wuhan, China; ^5^Department of Human Resources, Zhongnan Hospital of Wuhan University, Wuhan, China; ^6^Department of General Practice, Wuhan Donghu Hospital, Wuhan, China; ^7^Department of Radiation and Medical Oncology, Zhongnan Hospital of Wuhan University, Wuhan, China; ^8^Hubei Key Laboratory of Tumor Biological Behaviors, Wuhan, China; ^9^Hubei Cancer Clinical Study Center, Wuhan, China

**Keywords:** COVID-19, IL-2R, IL-6, liver injury, prognosis

## Abstract

The novel coronavirus disease 2019 (COVID-19), caused by severe acute respiratory syndrome coronavirus 2 (SARS-CoV-2), has spread worldwide, and the WHO declared it a pandemic on March 11, 2020. Clinical characteristics and epidemiology features of patients infected with SARS-CoV-2 have been explored in the previous study. However, little is known about the combinative association of liver dysfunction and abnormal interleukins (ILs) in severe patients with COVID-19. This study was designed to estimate whether liver dysfunction and abnormal ILs could predict the severity of COVID-19. This study integrated liver function data and ILs data in patients with COVID-19 and found that liver injury and two ILs, interleukin-2 receptor (IL-2R) and interleukin-6 (IL-6), were closely related to the prognosis of patients with COVID-19. This study may give more exact information to clinicians about the prognosis of patients with COVID-19. In addition, this correlational study between liver disorder and ILs may provide a new vision to diagnosis and treatment in patients.

## Introduction

The novel coronavirus disease 2019 (COVID-19), caused by severe acute respiratory syndrome coronavirus 2 (SARS-CoV-2), has spread worldwide and the WHO declared it a pandemic on March 11, 2020 (1). As of December 27, 2020, the number of SARS-CoV-2 cases globally had eclipsed 8,06,78,170, mainly exceeding the total number of SARS cases during the 2003 epidemic and more than 17,63,106 people had now died. The outbreak of COVID-19 has put health authorities on high alert in China and across the globe.

Clinical characteristics and epidemiology features of patients infected with SARS-CoV-2 have been explored in many previous studies (2, 3). COVID-19 usually presents with fevers and/or upper and/or lower respiratory symptoms, which may develop into multiorgan failure in severe patients (4). Liver enzymes are elevated in 14–53% of patients hospitalized with COVID-19. Although the degree of elevation is usually mild, severe elevations in aminotransferase levels have been reported (5). Liver dysfunction is vital because elevated liver enzymes may be associated with an increased risk of mortality (6). The exact mechanism driving COVID-19-related liver injury remains unclear and several mechanisms should be explored. Moreover, some studies indicated that the severity of illness was often related to liver dysfunction in patients with COVID-19 (7, 8). We focus on liver dysfunction to explore inner influence in patients with COVID-19. Meanwhile, abnormal interleukins (ILs) were detected in patients with severe SARS-CoV-2 compared with those with milder infections (9, 10). However, little is known about the combinative association of liver dysfunction and abnormal ILs in severe patients with COVID-19. Therefore, this study retrospectively investigated the potential relationship between COVID-19-associated liver injury and the disorder of ILs. Combining these two factors, liver injury and ILs disorder may give more exact information to clinicians about the prognosis of patients with COVID-19. In addition, this correlational study between liver disorder and ILs may provide a new vision to improve the early diagnosis and treatment in patients with COVID-19.

## Materials and Methods

### Study Population

This study was conducted on 151 hospitalized patients in Wuhan Leishenshan Hospital, P R China from February 8, 2020, to March 1, 2020. Records of the patient including clinical and laboratory data that were extracted and used for analysis. This study was approved by the Ethics Committee of Zhongnan Hospital of Wuhan University. None of the patients had underlying chronic liver disease. Moreover, all the complications that affected the expression of ILs, such as diabetes, cardiovascular disease, cerebrovascular disease, and kidney damage, were excluded from our study population.

### Ethics Committee Approval Stating

This study was reviewed and approved by the Ethics Committee of Zhongnan Hospital of Wuhan University. The Ethics document has been uploaded as a supplementary file.

### Data Collection

All the electronic medical records of 151 patients with confirmed COVID-19 were reviewed and the demographic characteristics, clinical data, and outcomes were collected. The majority of the clinical data used in this study was collected on admission at baseline. According to the guidelines for the diagnosis and treatment of novel coronavirus (2019-nCoV) infection by the National Health Commission (Trial Version 5), at the time of hospitalization, patients with one of respiratory rate > 30 breaths/min, oxygen saturation (SpO_2_) <93% on room air, or oxygen tension (PaO_2_)/fraction of inspiration O_2_(FiO_2_) ≤ 300 mm Hg were classified as severe cases and the others were classified as non-severe cases. Initial laboratory investigations included complete blood count, coagulation function, and routine biochemical and liver function tests. Serial monitoring of the laboratory profile was performed according to the clinical progress of individual patients.

### Biochemical Parameters

Alutamic-pyruvic transaminase (ALT), aspartate aminotransferase (AST), total protein (TP), albumin (Alb), serum alkaline phosphatase (ALP), gamma-glutamyl transpeptidase (γ-GGT), total bilirubin (TBil), direct bilirubin (DBil), indirect bilirubin (DBil), total bile acid (TBA), creatinine (Cre), and ureophil (urea) were obtained retrospectively from liver and kidney function test from the patients with COVID-19.

### Inflammatory Cytokines and Procalcitonin

Inflammatory cytokines and procalcitonin (PCT) were determined by Cobas 8000 (Roche, Basel, Switzerland) according to the specifications of the manufacturers.

### Statistical Analysis

All the statistical analyses were performed by SPSS software (version 19.0, IBM Corporation, New York, USA). Continuous data are presented as mean ± SD and categorical data are expressed as the proportions. The quantitative variables were compared by the Student's *t*-test or the Mann–Whitney *U* test, when appropriate. Meanwhile, the categorical variables were analyzed by the chi-squared test or the Fisher's exact test, as appropriate. Pearson's correlation test was used to determine the association between the two groups. The multivariate Cox proportional hazards model was also performed to the above variables. The receiver operating characteristic (ROC) curve generated was used to determine whether the aforementioned significant factors had diagnostic value. *p* < 0.05 was considered as statistically significant.

## Results

### Demographics and Clinical Characteristics

In this study, a total of 151 patients with COVID-19 (65 females and 86 males) were finally included and retrospectively analyzed. The characteristics of all the patients are shown in Table 1. The mean age was 62.7 years (range, 26–93 years) and 98 (64.9%) patients were older than 60 years. The most commonly initial symptoms were fever (*n* = 95, 62.9%), cough (*n* = 78, 51.7%), dyspnea (*n* = 48, 31.8%), fatigue or myalgia (*n* = 33, 21.9%), and digestive symptoms (*n* = 7, 4.6%). The median time from symptom onset to admission was 18.4 ± 10.7 days, whereas 32 (21.2%) patients developed high fever (body temperature ≥ 39.0°C). Of these 151 patients, more than half (*n* = 96, 63.6%) of patients received antiviral therapy including arbidol hydrochloride capsules and oseltamivir phosphate capsules. As of March 30, 2020, 5 patients died and 131 patients were discharged from the hospital, while 15 patients were still hospitalized. In the entire cohort, 22 patients developed acute respiratory distress syndrome (ARDS) and 21 patients were admitted to the intensive care unit (ICU), while 19 patients received mechanical ventilation. Of particular note, all the dead patients had developed ARDS and received mechanical ventilation.

**Table 1:** Demographic characteristics of patients with COVID-19.

**Study population**	**No. (%)**
**No. of patients**	151
≥60	98 (64.9%)
<60	53 (35.1%)
**Gender**	
Female	65 (43.0%)
Male	86 (57.0%)
**Mean time of illness onset to admission, days**	18.4 ± 10.7
**Highest temperature (** **°** **C)**	
≥39.0	32 (21.2%)
<39.0	119 (78.8%)
**Initial symptoms**	
Fever	95 (62.9%)
Cough	78 (51.7%)
Dyspnea	48 (31.8%)
Fatigue or myalgia	33 (21.9%)
Digestive symptoms	7 (4.6%)
**Clinical classification**	
Mild/Common	29/60 (58.9%)
Severe/Critical	50/12 (41.1%)
**Therapy**	
Antiviral	96 (63.6%)
Antibiotic	71 (47.0%)
Antioxidant	34 (22.5%)
Hormone	24 (15.9%)
Resochin	14 (9.3%)
**Clinical outcomes**	
ICU admission	21 (13.9%)
ARDS	22 (14.6%)
Discharged	131 (86.8%)
Died	5 (3.3%)
Hospitalization	15 (9.9%)

*Data are presented as n (%) or n/N (%), where N is the total number of patients in a group*.

*COVID-19, coronavirus disease 2019; ICU, intensive care unit; ARDS, acute respiratory distress syndrome*.

### Clinical Outcomes in Patients With Different Classification

All the patients were divided into 2 groups. As shown in Table 2, the severe group patients consisted of 30 women and 32 men with a mean age of 64.0 years, whereas the non-severe group patients composed of 50 women and 39 men with a mean age of 61.8 years with no statistical significance. Moreover, there was no significant difference between the two groups with respect to the highest temperature, initial symptoms, and comorbidities (*p* > 0.05 for each). Compared with nonsevere patients, patients with severe illness had a longer course of disease days (20.1 vs. 17.2 days, *p* < 0.05). In addition, severe patients were more likely to be treated with antibiotics and hormone therapy (34/89 vs. 37/62, *p* = 0.009; 7/89 vs. 17/62, *p* = 0.001), which may be due to more severe inflammatory reaction and bacterial infection occurred in severe patients. During follow-up, the proportion of patients who developed ARDS were higher in a severe group than in the non-severe group (5/89 vs. 17/62, *p* = 0.0002). In the severe group, a total of 4 patients died, 6 patients were discharged, 18 patients were admitted to ICU, and 52 patients remained hospitalized, while only 1 patient died, 50 patients discharged, 3 patients were admitted to ICU, and 38 remained hospitalized in the non-severe group (*p* < 0.01 for each). These results showed a positive correlation between clinical types and the prognosis of patients with COVID-19.

**Table 2:** Clinical characteristics among patients with different classification.

**Clinical characteristics**	**No-severe (*n* = 89)**	**Severe (*n* = 62)**	***P***
Age, mean ± SD, y	61.8 ±13.8	64.0 ± 13.9	0.17
Female/Male	50/39	30/32	0.35
Highest temperature mean ± SD, °C	38.0 ± 0.9	38.3 ± 0.9	0.02
Course of disease, mean ± SD, days	17.2 ± 10.9	20.1 ± 10.1	<0.05
**Initial symptoms**			
Fever	54	41	0.49
Cough	48	30	0.50
Dyspnea	27	21	0.65
Fatigue or myalgia	19	14	1.00
Digestive symptoms	6	1	0.14
**Therapy**			
Antiviral	54	42	0.37
Antibiotic	34	37	0.009
Antioxidant	17	17	0.23
Hormone	7	17	0.001
Resochin	9	5	0.67
**Clinical outcomes**			
ARDS	5	17	0.002
ICU admission	3	18	<0.01
Discharged	50	6	<0.01
Died	1	4	<0.01
Hospitalization	38	52	<0.01

*ICU, intensive care unit; ARDS, acute respiratory distress syndrome*.

### Combined With Hypercytokinemia and Liver Dysfunction in Severe or Critically Ill Patients

In the terms of laboratory findings, severe patients compared with the non-severe patients showed higher blood levels of inflammatory ILs in Table 3. Two inflammatory ILs, interleukin-2 receptor (IL-2R) (*p* = 0.01) and interleukin-6 (IL-6) (*p* = 0.001), were found to have relationships with inferior prognosis in patients with COVID-19. At the same time, an infection-related factor, PCT, was detected that was significantly correlated with the severity of COVID-19 (*p* = 0.02). Besides, biochemical indicators were also analyzed. A total of 5 liver injury-related factors, ALT (*p* < 0.01), AST (*p* < 0.01), TBil (*p* < 0.01), DBil (*p* < 0.01), and IBil (*p* < 0.01), were identified to be noteworthy prognostic factors in COVID-19 disease. Other biochemical indicators were not related to the severity of COVID-19.

**Table 3:** Clinical characteristics and initial laboratory indices among patients with different classification.

**Laboratory findings**	**No-Severe (*n* = 89) mean ± SD**	**Severe (*n* = 62) mean ± SD**	***P***
**Inflammatory cytokines**,			
IL-1β, pg/ml… 13 ± 4.6… 12 ± 3.2 0.44			
IL-2R, U/ml	486.8 ± 561.5	704.5 ± 453.8	<0.01
IL-8, pg/m …1 ± 1.1 … 4 ± 2.5 … 0.07			
TNF-α, pg/ml … 22 ± 24.7 … 21 ± 18.5 … 0.22			
IL-10, pg/ml … 4 ± 4.5 … 6 ± 5.1 … 0.21			
IL-6, pg/ml … 11 ± 6.7 … 24 ± 4.4% … <0.001			
**PCT, ng/ml**	0.1 ± 0.2	0.7 ± 2.3	0.02
**Biochemical**,			
ALT, IU/L	28.3 ± 28.3	107.1 ± 86.6	<0.01
AST, IU/L	23.3 ± 16.7	150.3 ±45.8	<0.01
TP, g/L	72.4 ± 67.2	62.2 ± 7.0	0.92
Alb, g/L	37.0 ± 4.8	33.2 ± 5.0	1
ALP, IU/L	77.3 ± 27.4	78.0 ± 31.3	0.44
γ-GGT, IU/L	41.2 ± 35.9	47.5 ± 39.5	0.16
TBil, μmol/L	9.6 ± 5.0	12.3 ± 5.5	<0.01
DBil, μmol/L	3.7 ± 2.3	5.3 ± 3.3	<0.01
IBil, μmol/L	5.6 ± 2.7	7.1 ± 3.1	<0.01
TBA,μmol/L	6.9 ± 8.2	6.6 ± 7.4	0.59
Cre, μmol/L	67.3 ± 22.8	101.8 ±28.6	0.12
Urea, mmol/L	5.6 ± 2.2	8.0 ± 11.5	0.06

*IL, interleukin; TNF, tumor necrosis factor; PCT, procalcitonin; ALT, glutamic pyruvic transaminase; AST, glutamic oxalacetic transaminase; TP, total protein; Alb, albumin; ALP, serum alkaline phosphatase; γ-GGT, gamma-glutamyl transpeptidase; TBil, total bilirubin; DBil, direct bilirubin; IBil, indirect bilirubin; TBA, total bile acid; Cre, creatinine; Urea, ureophil*.

### Pearson's Correlation Coefficients Between IL-2R, IL-6, PCT, and Liver Function Indexes

To further verify the relationship between cytokines and liver injury, Pearson's correlation was applied between IL-2R, IL-6, PCT, and liver function indexes. Inflammatory cytokines, IL-2R and IL-6, were positively correlated with ALT (*p* = 0.01, *p* = 0.01) and AST (*p* < 0.01, *p* < 0.01). Nevertheless, TBil, DBil, IBil, and PCT had no significant correlation as shown in Table 4.

**Table 4:** Pearson's correlation coefficients between IL-2R, IL-6, PCT, and liver function indexes.

**Variables**		**ALT**	**AST**	**TBil**	**DBil**	**IBil**
IL-2R	r	0.335	0.243	0.251	0.363	0.481
	*P*-value	0.010	0.010	0.068	0.078	0.086
IL-6	r	0.432	0.543	0.651	0.563	0.331
	*P*-value	<0.001	<0.001	0.161	0.065	0.0733
PCT	r	0.437	0.643	0.551	0.582	0.361
	*P*-value	0.061	0.107	0.159	0.203	0.575

*IL, interleukin; PCT, procalcitonin; ALT, glutamic pyruvic transaminase; AST, glutamic oxalacetic transaminase; TBil, total bilirubin; DBil, direct bilirubin; IBil, indirect bilirubin; IL-2R, interleukin-2 receptor; IL-6, interleukin-6*.

### Receiver Operating Characteristic Analysis

**Figure 1:**
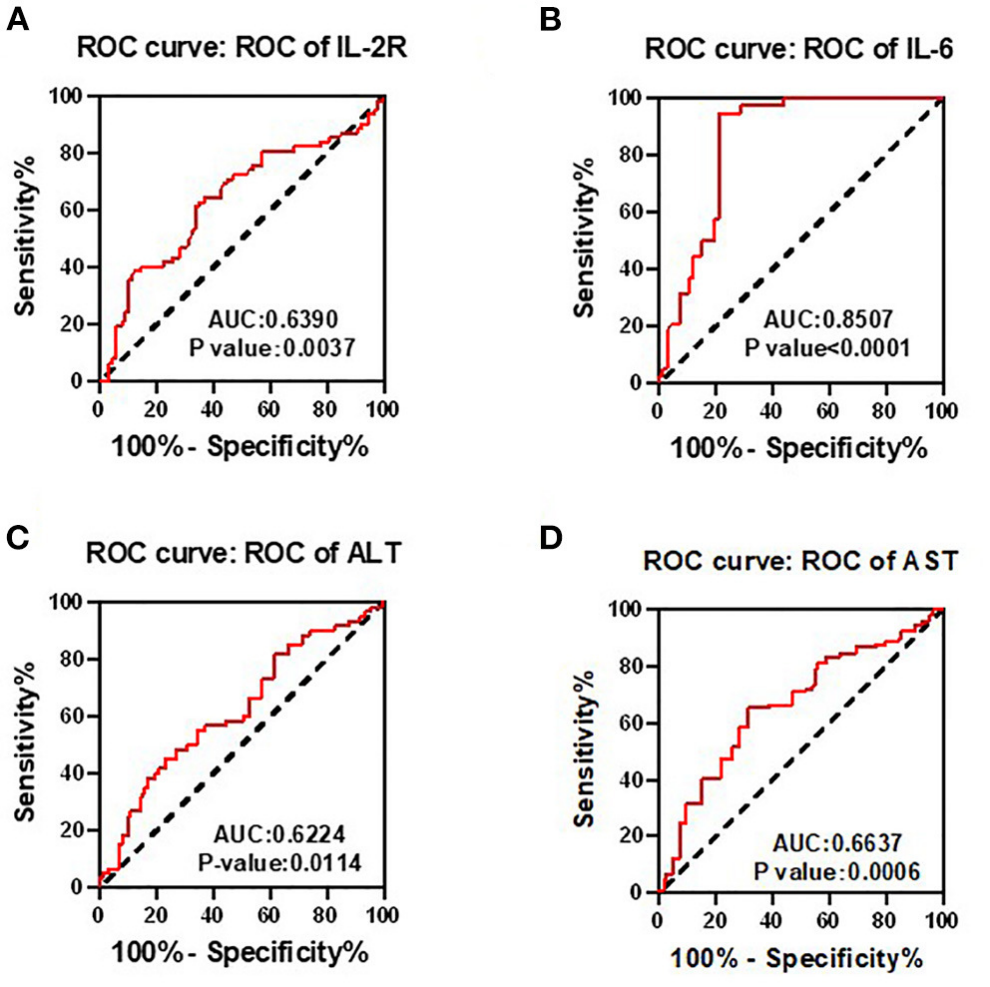
Receiver operating characteristic (ROC) analyses were used to determine whether the interleukin-2 receptor (IL-2R), interleukin-6 (IL-6), ALT, and AST had diagnostic value. Area under the curve (AUC) of IL-2R **(A)**, IL-6 **(B)**, ALT **(C)**, and AST **(D)** was 0.639 (*p* = 0.0037), 0.8507 (*p* < 0.0001), 0.6224 (*p* = 0.0021), 0.6637 (*p* = 0.0006), respectively. Therefore, IL-2R, IL-6, ALT, and AST could be identified as potential prognostic biomarkers in patients with COVID-19.

To determine whether the aforementioned significant factors had diagnostic value, ROC analysis was performed. As showed in Figure 1, area under the curve (AUC) of IL-2R, IL-6, ALT, and AST was 0.639 (*p* = 0.0037), 0.8507 (*p* < 0.0001), 0.6224 (*p* = 0.0021), 0.6637 (*p* = 0.0006), respectively. Therefore, IL-2R, IL-6, ALT, and AST could be identified as potential prognostic biomarkers in patients with COVID-19.

## Discussion

Hepatic injuries occur in a significant subpopulation of patients with COVID-19, particularly those with moderate-to-severe illness (5, 8). However, the underlying mechanism of liver injury has not been conclusively determined in patients with COVID-19. Viral-induced cytopathic effect and potential hepatotoxicity from the therapeutic drugs are the two most potential underlying mechanisms for liver injury in patients with COVID-19. In addition, acute exacerbation of chronic hepatitis in the course of the disease or hypoxic liver injury caused by long-term hypoxia may also be an important mechanism of liver injury in patients with COVID-19 (5, 7). In this study, the integrated relationship between liver injury and ILs, IL-2R, and IL-6, were explored for the first time.

In this study, the demographic characteristics and detailed laboratory examination indices were analyzed in patients with COVID-19. In terms of laboratory findings, critical patients compared with noncritical patients showed higher blood levels of ILs. IL-2R (*p* = 0.01) and IL-6 (*p* = 0.001) were found to have relationships with inferior prognosis in patients with COVID-19. During immune activation, CD25 is expressed by T cells and its soluble form (sCD25), also known as IL-2R, was released into the bloodstream (11). Hongyan Hou et al. found that IL-2R was a significant prognostic factor for patients with COVID-19, which was consistent with this study (12). We speculate that inhibition of IL-2R may also inhibit inflammatory storms that will provide a new strategy for the treatment of patients with COVID-19. IL-2R blockade therapy may be a promising treatment strategy for patients with COVID-19. IL-6 was detected to host defense against infections and tissue injuries (13). However, exaggerated, excessive synthesis of IL-6, while fighting SARS-CoV-2, leads to an acute severe systemic inflammatory response known as cytokine storm (14). Remarkable beneficial effects of IL-6 blockade therapy using a humanized anti-IL-6 receptor antibody, tocilizumab, were recently observed in patients with cytokine release syndrome complicated by T-cell engaged therapy (15, 16). This study confirmed that high IL-6 was associated with the poor prognosis of patients with COVID-19. Thus, we further demonstrated that IL-6 blockade might constitute a novel therapeutic strategy for severe patients with COVID-19.

Proinflammatory ILs play an important role in the immune reaction during virus infections and can predict the severity of clinical outcomes (17). According to a study by Min, 308 patients with COVID-19 showed that severe cases had a higher level of proinflammatory factors at admissions such as IL-2R, IL-6, IL-8, IL-10, and TNF-α. Hyperinflammatory responses in severe cases of COVID-19 may correlate with the clinical outcomes and prognosis (18). Lin et al. also found that long-term infection with SARS-CoV-2 may result in an increase in the cytokines IL-6, TNF-α, IFN-γ, IL-2, IL-4, and IL-10 (19). Meanwhile, many previous studies have confirmed that liver dysfunction was closely related to the prognosis of patients with COVID-19 (5, 8). The main serum indexes of liver function including ALT, AST, TBIL, DBil, and IBil could maintain the stability of hepatocytes under a normal intracellular environment (20). These indexes of liver function in severe cases of COVID-19 showed significant elevation compared with that in mild cases in this study. These results indicate that the liver may be one of the main target organs of SARS-CoV-2 infection. This study integrated liver function data and ILs data in patients with COVID-19 and found that they were closely related to the prognosis of patients with COVID-19. This study will provide a new prognostic factor for patients with COVID-19. However, the underlying mechanism between liver injury and ILs still needs further basic study.

## Conclusion

In conclusion, our present findings suggested that liver injury and ILs disorder could be independent prognostic factors for patients with COVID-19. We propose that blockade therapy of IL-6 and IL-2R will be a promising treatment strategy for patients with COVID-19. Liver dysfunction has many clinical symptoms and laboratory indexes. However, considering the limited hospital conditions at that urgent time and the retrospective nature of this study, we only have sufficient data of blood samples for analysis. Large-scaled prospective trials are still warranted to verify our results. Besides, our study background was in the early stage of the outbreak of COVID-19 pneumonia when variants of COVID-19 had not happened and vaccination was not yet available. We could not discuss the clinical recommendations or implications in the context of more cases with variants of COVID-19 and patients with vaccination. This is another flaw of our study. Up-to-date relevant data need to be collected.

## Data Availability Statement

The raw data supporting the conclusions of this article will be made available by the authors, without undue reservation.

## Ethics Statement

The studies involving human participants were reviewed and approved by the Ethics Committee of Zhongnan Hospital of Wuhan University. The patients/participants provided their written informed consent to participate in this study. Written informed consent was obtained from the individual(s) for the publication of any potentially identifiable images or data included in this article.

## Author Contributions

ML contributes to the study design, data interpretation, and original draft preparation. KL, JY, YC, and QT contribute to the study identification, data extraction, and statistical analysis. All authors revised and approved the final version of the manuscript.

## Funding

This study was supported by a grant from the Special Fund for Central Universities in China (no. 413000260).

## Conflict of Interest

The authors declare that the research was conducted in the absence of any commercial or financial relationships that could be construed as a potential conflict of interest.

## Publisher's Note

All claims expressed in this article are solely those of the authors and do not necessarily represent those of their affiliated organizations, or those of the publisher, the editors and the reviewers. Any product that may be evaluated in this article, or claim that may be made by its manufacturer, is not guaranteed or endorsed by the publisher.
